# Flower colour contrast, ‘spectral purity’ and a red herring

**DOI:** 10.1111/plb.13767

**Published:** 2025-01-27

**Authors:** C. J. van der Kooi, J. Spaethe

**Affiliations:** ^1^ Groningen Institute for Evolutionary Biology University of Groningen Groningen The Netherlands; ^2^ Department of Behavioral Physiology and Sociobiology University of Würzburg Würzburg Germany

**Keywords:** flower colour, Pollination, spectral purity, spectral sensitivity, vision modelling, visual ecology

## Abstract

Nature offers a bewildering diversity of flower colours. Understanding the ecology and evolution of this fantastic floral diversity requires knowledge about the visual systems of their natural observers, such as insect pollinators. The key question is how flower colour and pattern can be measured and represented to characterise the signals that are relevant to pollinators. A common way to interpret flower colours is using animal vision models that incorporate the spectral sensitivity of a focal observer (e.g. bees). These vision models provide a measure of colour contrast, which represents the perceived chromatic difference between two objects, such as a yellow flower against green leaves. Colour contrast is a behaviourally and physiologically validated proxy for relative conspicuousness of a stimulus. A growing number of studies attempt to interpret flower colouration through parameters that are grafted on to principles of human colour perception. A perpetuating measure to describe floral colours is via saturation, which is a metric in human perception describing a certain aspect of colourfulness and is, in pollination literature, often referred to as ‘spectral purity’. We caution against the concept, calculation and biological interpretation of ‘spectral purity’ and similar measures that rest on an anthropocentric view, because it does not represent the diversity and complexity of animal visual systems that are the natural observers of flowers. We here discuss the strengths and weaknesses of common ways to interpret flower colouration and provide concrete suggestions for future colourful research.

## Graphical abstract

We discuss the strengths and weaknesses of common ways to calculate the visual conspicuousness of flowers to pollinators using animal vision models.

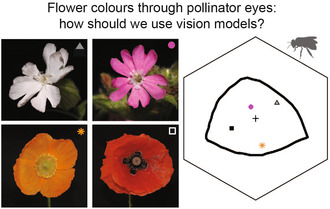

## INTRODUCTION

Earth's natural flora is resplendent with flower colours. The mesmerising diversity of floral colours has fascinated mankind for centuries. Aristotle commented on how colours aid a flower's visibility to insects, and Darwin's writing on how flowers attract pollinators is still inspirational for many people today. How do the optical properties of flowers evolve to attain visual signals that are attractive to pollinators? This key question in pollination and flower biology can be addressed from different angles. For example, molecular biological studies have revealed the diversity in genetic pathways and transcription factors that determine flower pigmentation (reviewed by Grotewold 2006; Rausher 2008). Optical studies have illuminated how cell structure, interior stratification and pigment localisation of flowers together create the visual signal (reviewed by van der Kooi *et al*. 2019).

Addressing the ultimate aspects of what makes floral visual signals attractive to pollinators requires detailed knowledge of the visual systems of common pollinators, such as insects. A pollinator's approach to a flower is a series of behavioural reactions that might be triggered by different signals. From a distance, which can be before the animal is able to visually distinguish the flower, floral scent can trigger a behavioural approach. Long‐range visual detection by insects commonly occurs through achromatic (intensity‐based) cues, whereas colour vision occurs at short range (Giurfa *et al*. 1996, 1997; Jezeera *et al*. 2021; Meena *et al*. 2021; van der Kooi & Kelber 2022). For example, a circular flower with a diameter of 5 cm can be seen by honeybees at 50 cm through achromatic cues, and at 19 cm based on colour information (after Giurfa *et al*. 1996). When the insect has approached the flower, short‐range navigation and foraging behaviour within the flower can be mediated by within‐flower colour patterns (Hempel De Ibarra *et al*. 2015; Richter *et al*. 2023).

**Fig. 1:**
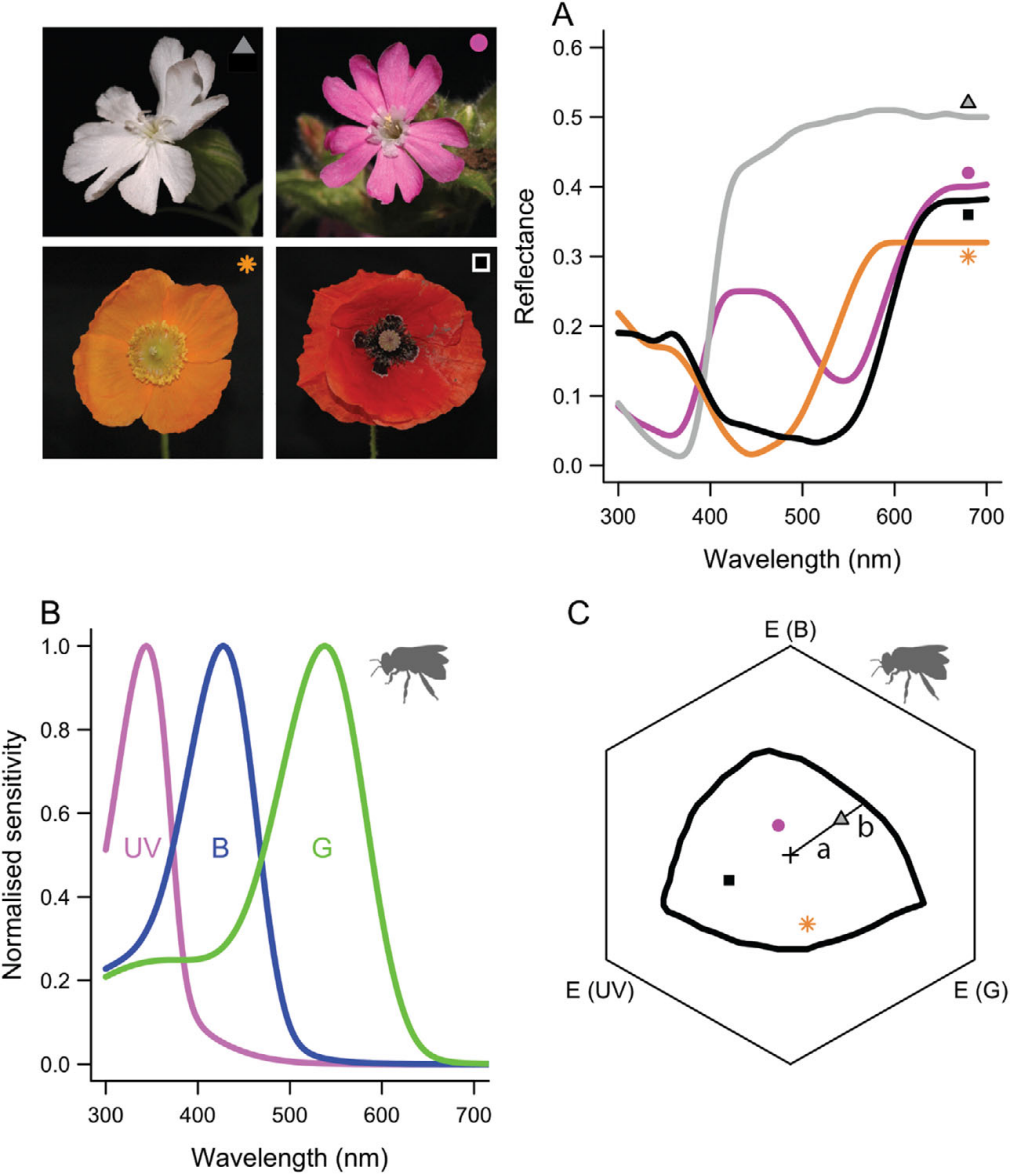
Bee visual space with four exemplary flowers and calculation of colour contrast to the background and ‘spectral purity’ (after van der Kooi & Spaethe 2022). (A) Reflectance spectra of four exemplar flowers, which pictures are shown on the left. Species shown: *Silene latifolia–alba* (grey triangle), *Silene dioica* (magenta circle), *Papaver* (*Meconopsis*) *cambrica* (orange star) and European *Papaver rhoeas* (black square). (B) Spectral sensitivity of honeybees, showing peak sensitivities in the ultraviolet (UV), blue (B) and green (G) wavelength ranges (Peitsch *et al*. 1992). (C) The hexagon colour space showing the four exemplary species from A. The achromatic centre of the hexagon, which represents the (green) background colour to which the photoreceptors are adapted, is depicted with a ‘+’ sign. The plotted black boundary line is after Chittka (1992). ‘Spectral purity’ (as per Lunau 1990; Rohde *et al*. 2013) is calculated as the relative distance of a stimulus to the monochromatic line, that is, spectral purity = *a*/(*a* + *b*); where *a* is the flower's colour contrast to the background, and *b* is the distance between the flower and the boundary of the visual space. The colour contrast between two flowers is represented by their Euclidean distance in visual space. Different corners of the hexagon (marked with ‘E’) represent relative excitation of the different photoreceptor types. The honeybee silhouette was obtained from phylopic.org.

An established way to interpret flower colours is through animal vision models that incorporate the reflectance spectra of flowers and the background, the illumination conditions, and the spectral sensitivity of a focal observer (e.g. bees). Examples of insect vision models are the colour triangle, colour opponent coding model, colour hexagon and receptor noise‐limited model (Backhaus 1991; Chittka 1992; Vorobyev & Osorio 1998; Kelber *et al*. 2003). Vision models can visualise where different flower colours cluster in different parts of a pollinator's ‘visual space’ (Fig. 1). Virtually all bees have highly similar colour vision, with peak sensitivities in the ultraviolet, blue and green wavelength range (Fig. 1B, Peitsch *et al*. 1992). The position of a flower in the visual space is determined by the relative excitation of different photoreceptor types. For example, the light reflected by *Silene dioica* flowers most strongly excites the blue photoreceptor type, which is why in visual space it is closest to the blue corner (Fig. 1C). Depending on the context, different vision models have their strengths and weaknesses (for reviews, see Kelber *et al*. 2003; Kemp *et al*. 2015). Vision models allow one to quantify the difference or similarity between two stimuli (i.e. colour contrast), such as a yellow flower against a green leaf environment or between two different flowers. Colour contrast as obtained through animal vision models is a behaviourally and physiologically validated proxy for stimulus conspicuousness for many animals, including insects and vertebrates (Giurfa *et al*. 1997; Spaethe *et al*. 2001; Kelber *et al*. 2003; Dyer & Chittka 2004; Kemp *et al*. 2015; Vasas *et al*. 2019).

Attempts have been made to discern the relative importance of different components that constitute the colour difference between two stimuli. The colour contrast between two stimuli is generally determined by their hue and chroma/saturation of the stimuli. Hue is the attribute, or categorical description, of the dominant shade or tone of a flower, for example blue or yellow. Hue is what is most often meant when discussing ‘colour’. Chroma and saturation represent metrics of human colour vision that describe different aspects of ‘colourfulness’ of a stimulus in human perception; for example, pink is of lower chroma/saturation than red. Similarly, pastel colours are of lower chroma/saturation than vibrant, bold colours. To apply the concept of chroma/saturation from human vision to flower colours, Lunau (1990) coined the idea of floral ‘spectral purity’, which he defined as the relative distance of a flower's point in bee visual space to the boundary of the visual space. The boundary of a visual space is composed of artificial stimuli with maximum saturation (i.e. monochromatic lights, see the black line in Fig. 1). Fig. 1 shows how colour contrast and ‘spectral purity’ are calculated. Lunau's approach has been adopted in numerous other studies (e.g. Rohde *et al*. 2013; Shrestha *et al*. 2014; Koethe *et al*. 2022). Initially, ‘spectral purity’ was calculated using the colour triangle (Lunau 1990), but it was later calculated with Chittka's (1992) hexagon model. Although other behaviourally validated vision models have been developed since (e.g. Vorobyev & Osorio 1998; Endler & Mielke 2005), ‘spectral purity’ is virtually always calculated using the colour hexagon.

We previously articulated that the concept, calculation and biological interpretation of floral ‘spectral purity’ and measures to quantify ‘saturation’ or ‘chroma’ in insect colour perception rest on dubious foundations, and that the quest for quantifying floral ‘spectral purity’ is founded on an anthropocentric view that does not represent the diversity and complexity of animal visual systems that are the natural observers of flowers (van der Kooi & Spaethe 2022). Lunau & Dyer (2024) challenge some of our reasoning. Here, we elaborate on our previous claims, with the aim to share our views on the strengths and weaknesses of pollinator vision modelling with the flower and pollination community.

## HUE AND ‘SPECTRAL PURITY’—COMPARING APPLES AND ORANGES

Lunau & Dyer (2024) raise a few important points; for instance, that it is poorly understood how the direction (sign) of colour differences between two stimuli (e.g. a flower colour pattern) determines the behavioural response by insect pollinators. We furthermore have sympathy for the authors' stance that not all behaviour lends itself to simple clarity, black and white, every time. Several other statements deserve to be echoed. For example, we could not agree more with their points that flower colour and animal colour vision are complex, that colour should not be considered a ‘trait’ but rather is a perception, and that we need to better study the optical properties of flowers for a proper understanding of the visual signalling between flowers and pollinators.

Human‐centric terminology can be applied to convey the details of the optical intricacies of flowers, but that does not mean that pollinators perceive colour attributes similarly to humans (Kemp *et al*. 2015). The *type* of pigment largely determines the floral hue, and the *amount* (or density) of pigment largely determines to humans the colourfulness of a flower (van der Kooi 2021). Concentrating pigment in the epidermal layer at the side of viewing can be a cost‐effective way to further enhance visual contrast (van der Kooi *et al*. 2016; Fan *et al*. 2024). Although changes in both hue and chroma/saturation will affect a flower's colour contrast, it is meaningless to quantify the contribution of chroma/saturation to overall colour contrast when two stimuli have completely different hues. As an example, addressing a question such as ‘is the colour contrast between purple and orange flowers caused by the difference in hue or more because they differ in “spectral purity”?’ is comparing plums and oranges. Deploying human‐centric terms to understand floral visual signalling is even more questionable given that the visual systems of humans are very different from that of natural pollinators, especially insects. There is no psychophysical or behavioural evidence to support the premise that an insect weighs human‐defined characteristics of spectral information, such as hue, chroma or saturation. Therefore, it is not ecologically meaningful nor justified to quantify the importance of hue versus ‘spectral purity’ in creating colour contrast.

## CONCEPTUAL CONCERNS WITH CALCULATING ‘SPECTRAL PURITY’

Setting aside the wobbly rationale of attempting to discern the importance of ‘spectral purity’ to the visibility of flowers, there are at least five key issues regarding the theory, calculation and interpretation of measures for ‘spectral purity’ (van der Kooi & Spaethe 2022).

1. A dearth of quality behavioural evidence that supports the premise that pollinators perceive and respond to ‘spectral purity’ or any other measure that isolates the effect of human‐perceived chroma/saturation of a visual stimulus.
2. There are multiple weaknesses in the calculation procedure of ‘spectral purity’. First, there is no evidence supporting the drawing of a lower border of the visual space and connecting the 700 and 300 nm corners. Adding a hypothetical lower border is convenient for calculations, but not grounded in evidence. The electromagnetic spectrum, which, by definition, is linear, is in essence made circular with this line. Second, by making the visual space virtually circular, one inflates the weight that is given to small variation. Third, calculating ‘spectral purity’ creates artefacts for flowers with high reflectance in the ultraviolet and low reflectance in the 400–600 nm wavelength range, such as UV‐reflecting red flowers (Lunau *et al*. 2011; van der Kooi & Stavenga 2019; Chen *et al*. 2020; León‐Osper & Narbona 2022), because of issues with the monochromatic line in the UV‐corner of the bee visual space (van der Kooi & Spaethe 2022).
3. There is no quality behavioural or psychophysics evidence to support using the border of a visual space for calculations to derive a measure of ‘spectral purity’. The border of a visual space is composed of colours (loci) of artificial, monochromatic lights. It thus is a useful illustration of the breadth of an animal's visual space (Fig. 1) but should not be used for calculations. In addition, ‘spectral purity’ as defined by the relative distance of a flower stimulus to the boundary of the visual space (sensu Lunau 1990; Rohde *et al*. 2013) is calculated as if the effect is linear (also by Lunau & Dyer 2024), but there is no evidence to support the assumption that any such relationship, if it exists, is indeed linear. Actually, most sensorial processes exhibit a nonlinear stimulus response behaviour, because of the fact that the underlying sensory receptors exhibit sigmoidal‐like response properties, which also occur in colour vision (Olsson *et al*. 2015; Garcia *et al*. 2017).
4. Values for ‘spectral purity’ and colour contrast are extremely correlated. We reanalysed more than 700 reflectance spectra from in a total of seven studies and showed that for honeybees, bumblebees and stingless bees, colour contrast and ‘spectral purity’ are highly correlated (see Figure 3 in van der Kooi & Spaethe 2022). Correlation coefficients are between 0.86 and 0.99 and highly significant (*p* < 0.001 in all cases, corrected for multiple testing). There is scientific consensus that when two parameters are highly correlated, they should not be treated as independent measures.
5. Inclusion of additional calculations in vision models increases the risk of introducing false positives.

These five elements individually — and certainly altogether — invalidate using ‘spectral purity’ as a metric for flower colour visibility to insects. It is unfortunate, therefore, that Lunau and Dyer only thoroughly respond to one point (point 1 above) of the five different weaknesses we had previously articulated (van der Kooi & Spaethe 2022). Our critiques about issues with the calculation procedure, the lack of empirical data to support the calculations, extremely correlated values that are treated as independent, and that of introducing biases remain open. The pollination community would benefit from quality empirical data that disentangle the importance of different parameters for visual signalling. We will now address some of the points that Lunau and Dyer raised regarding behavioural evidence suggesting a role for ‘spectral purity’ and its correlation to colour contrast.

## WHAT TYPE OF DATA WOULD VALIDATE ‘SPECTRAL PURITY’ AS A MEASURE FOR FLOWER VISIBILITY TO POLLINATORS?

Lunau and Dyer provide a reinterpretation of old data, collected by Lunau and colleagues almost three decades ago (Lunau *et al*. 1996). We identify several fundamental concerns with the type of data, its reanalysis and interpretation. First, the use of innate antennation behaviour as a proxy for bee responses to visual stimuli in general is problematic, because antennation is a highly specific type of behaviour that may well be biased by innate (colour) preferences. The usage of antennation behaviour alone does not do justice to the complexity of behavioural processes of an insect's natural approach flight and the various visual processes that it entails (see Introduction). Although antennation can characterise specific interactions with plants or conspecifics (Staudacher *et al*. 2005; Goyret 2010), we caution against using any response based on antennation behaviour to represent the diverse and complex behavioural responses that bees exhibit towards (visual) stimuli. [Correction added on 3 February 2025, after first online publication: the first sentence of this paragraph is updated in this version.]

Second, it is unclear in the experiment by Lunau *et al*. (1996) that Lunau & Dyer (2024) have reanalysed whether the bees used colour vision or achromatic vision. Colour processing by honeybees and bumblebees occurs at much shorter distances than that at which they use achromatic cues (Dyer *et al*. 2008). The tested nectar guides had a dimension of 2 × 4 mm, meaning that to process the nectar guides' colours the maximum viewing distance is 0.8 cm and 1.9 cm for honeybees and bumblebees, respectively. When using achromatic contrast, bees have a much higher resolution and could detect the small markings from a longer distance (Giurfa *et al*. 1996, 1997; Jezeera *et al*. 2021; Meena *et al*. 2021). Unfortunately, the use of achromatic contrast was not addressed in the original publication (Lunau *et al*. 1996), meaning it might be possible that the bees used the achromatic channel during their decision‐making and any response had little or nothing to do with colour.

Third, it is unknown what background colour the bee photoreceptors are adapted to during antennation. Photoreceptor adaptation to the background is an essential part of bee colour vision (Neumeyer 1980; Chittka 1992; Giurfa *et al*. 1997; Spaethe *et al*. 2001). When a bee is physically so close that it can touch the stimulus, the bee's visual field is dominated by the overall colour of the target. It is currently unknown whether under such a scenario the photoreceptors are adapted to the overall background of the arena/habitat or whether they (partially) adapt to the dominant target colour.

Fourth, although we have no reason to doubt that for his many inspiring experiments Lunau tested thousands of bee approaches, we argue that it is incorrect to consider these approaches as independent visits, as seems to have occurred. Indeed, during behavioural experiments with bees, a single bee is often tested multiple times, which can be five, 10 or even more. Different visits by a single bee should not be treated as independent observations, because bees have their personal preferences that are known to shape the results of behavioural assays (Muller & Chittka 2012). Similarly, there may be a colony effect (Raine & Chittka 2007), meaning that a set of stimuli needs to be tested by bees from different colonies.

It is unfortunate that Lunau and Dyer do not report the number of biological replicates, the achromatic contrast values of any of their stimuli, what data were extracted from which study, and how they were together reanalysed, rendering it next to impossible to evaluate their new results. What would be needed to test the importance of ‘spectral purity’ in plant–pollinator signalling is a behavioural experiment where an effect of ‘spectral purity’ can be disentangled from colour contrast to the background and/or other stimuli. For example, a set of stimuli with a similar hue and similar colour contrast to the background but varying in ‘spectral purity’. It may be hard to design such colour stimuli that resemble natural floral colours, given the strong correlation of these different elements. Ideally, the experimental stimuli are tested for different types of insect, including non‐bee pollinators, for example, flies, moths and butterflies, in keeping with contemporary knowledge about the broad suite of animal taxa that do serve as flower visitors in nature.

## CHROMA, SATURATION AND ‘SPECTRAL PURITY’: WHAT DOES IT MEAN?

We previously articulated how the frequent inaccurate terminology to describe colourfulness of flowers (as perceived by humans) adds to the convoluted nature of floral ‘spectral purity’ and other human‐based metrics such as ‘chroma’ (van der Kooi & Spaethe 2022). We provided the formal definitions as per Wyszecki & Stiles (1982) and CIE International Lighting Vocabulary (see Table 1 in van der Kooi & Spaethe 2022). For example, we explained that the term ‘saturation’ should be applied only to describe colourfulness of *luminous* stimuli (e.g. light sources, monitors) and not to *reflected* light, such as that of flowers. As for ‘spectral purity’, we followed Lunau (1990), who defined it as follows: ‘*The spectral purity is defined to be minimal* (*0%*) *at the locus of the neutral point and maximal* (*100%*) *at the spectral locus. The spectral purity of flower dummy colours* (*P*) *was calculated as the distance from the locus of the neutral point* (*N*) *to the locus of the flower dummy colour* (*F*) *as related to the distance from the locus of the neutral point to the locus of a reference spectral colour of maximal spectral purity. As a reference spectral colour, the spectral locus of the respective corresponding wavelength* (*Fc*) *was taken*.’ Fig. 1 visualises how ‘spectral purity’ can be calculated using that definition. That very definition of ‘spectral purity’ that uses the monochromatic line was adopted by Lunau, Dyer and others in subsequent research (e.g. Rohde *et al*. 2013; Shrestha *et al*. 2014; Koethe *et al*. 2016, 2018).

Adding to the confusion, Lunau & Dyer (2024) now add yet an alternative way to describe ‘spectral purity’, namely distance of a flower stimulus to the achromatic centre, that is, the background, in pollinator visual space (after Lunau *et al*. 1996). That latter concept is, however, identical to what is widely considered to represent a flower's colour contrast to the background (Chittka 1992; Kelber *et al*. 2003; Chittka & Wells 2004; Dyer 2006; Kemp *et al*. 2015). We caution against introducing new terms for concepts, such as colour contrast to the background, that are thoroughly described in seminal papers and are well established in visual ecological and pollination literature. Furthermore, this alternative definition of ‘spectral purity’ that does not consider the monochromatic line (sensu Lunau & Dyer 2024) yields a different and sometimes conflicting value for ‘spectral purity’ as per the original definition (sensu Lunau 1990; Rohde *et al*. 2013). For example, *Papaver rhoeas* (black square) and *Silene dioica* (grey triangle) have a similar distance to the background but differ in their relative distance to the monochromatic line (see Fig. 1). These two flowers would thus have different ‘spectral purity’ values according to Lunau's first definition (sensu Lunau 1990; see also Rohde *et al*. 2013; Shrestha *et al*. 2014; Koethe *et al*. 2022) but have similar values for ‘spectral purity’ according to the alternative definition that Lunau & Dyer (2024) recently proposed.

## CONCLUDING REMARKS AND OUTLOOK

Currently, there is no behavioural or psychophysics evidence to suggest that floral ‘spectral purity’ or any other metric that attempts to isolate the effect of saturation/chroma using colour vision models plays a role in visual signalling to pollinators. The new interpretation of old data provided by Lunau and Dyer is interesting but unconvincing. There are many unknowns about insect vision in that context, achromatic contrast is disregarded, their definitions and calculations of ‘spectral purity’ are ambiguous, and the studied behaviour is of a highly specific type (antennation) that is not representative for the behavioural complexity of bees or other insects. In addition, multiple previously raised concerns as to the calculation procedure of ‘spectral purity’ remain unresolved (see: Conceptual concerns with calculating ‘spectral purity’). Established and behaviourally validated measures for flower visibility to pollinators currently are:

1. Colour contrast to the background, calculated as the Euclidean distance between a flower and the achromatic centre in a visual space (Chittka 1992; Giurfa *et al*. 1997; Spaethe *et al*. 2001; Kelber *et al*. 2003; Kemp *et al*. 2015). Colour contrast can be calculated using various colour vision models, which each have their strengths and weaknesses (for reviews see Kelber *et al*. 2003 and Kemp *et al*. 2015).
2. Colour contrast between flowers or within flowers in case of colour patterns, calculated as the Euclidean distance between two stimuli in a visual space (Chittka 1992; Kelber *et al*. 2003).
3. Achromatic contrast between flowers and the background. This is calculated as the absolute difference between the flower and background as mediated by the long‐wavelength (green) photoreceptor (Giurfa *et al*. 1996; Spaethe *et al*. 2001; reviewed by Kelber *et al*. 2003; van der Kooi & Kelber 2022).
4. Hue (dominant colour) can in specific cases be a relevant predictor, for example when pollinators have a clear colour preference or in the context of floral mimicry. In the colour hexagon, hue is defined by the direction (angle) of the vector formed by the straight line from the centre to the colour locus.

We applaud development of new, diligent, carefully designed behavioural assays on a range of floral visitors to better understand plant–pollinator visual signalling and flower colour evolution. Of particular interest are studies that investigate wavelength‐specific weighting by photoreceptors (Von Helversen 1972) and the interplay of intensity (‘brightness’) and colour perception, that is, the Bezold‐Brücke effect (Chittka 1992; Chittka & Wells 2004). Until that has been achieved, we should refrain from overparameterization in the use of vision models, and deploy pollinator vision models following the principle of parsimony, that is, do not multiply beyond necessity.

## AUTHOR CONTRIBUTION

Both authors contributed equally to this manuscript.

## FUNDING INFORMATION

This work is supported by Human Frontiers Science Program (RGP023/2023, https://doi.org/10.52044/HFSP.RGP0232023.pc.gr.168611) and AFOSR (FA8655‐23‐1‐7049).
